# Designing low-cost, accurate cervical screening strategies that take into account COVID-19: a role for self-sampled HPV typing

**DOI:** 10.1186/s13027-020-00325-4

**Published:** 2020-10-14

**Authors:** Kayode Olusegun Ajenifuja, Jerome Belinson, Andrew Goldstein, Kanan T. Desai, Silvia de Sanjose, Mark Schiffman

**Affiliations:** 1 OAUTHC, Ile Ife, Nigeria; 2 Women’s Health Institute, Cleveland Clinic, Cleveland, OH USA; 3 Centers for Vulvovaginal Disease, Washington, DC USA; 4 Division of Cancer Epidemiology and Genetics, National Cancer Institute, Clinical Epidemiology Unit, Clinical Genetics Branch, 9609 Medical Center Drive Room 6E544, Rockville, MD 20850 USA

**Keywords:** HPV, Cervical screening, Self-sampling, Triage, COVID-19

## Abstract

**Background:**

We propose an economical cervical screening research and implementation strategy designed to take into account the typically slow natural history of cervical cancer and the severe but hopefully temporary impact of COVID-19. The commentary introduces the practical validation of some critical components of the strategy, described in three manuscripts detailing recent project results in Asia and Africa.

The main phases of a cervical screening program are 1) primary screening of women in the general population, 2) triage testing of the small minority of women that screen positive to determine need for treatment, and 3) treatment of triage-positive women thought to be at highest risk of precancer or even cancer. In each phase, attention must now be paid to safety in relation to SARS-CoV-2 transmission. The new imperatives of the COVID-19 pandemic support self-sampled HPV testing as the primary cervical screening method. Most women can be reassured for several years by a negative test performed on a self-sample collected at home, without need of clinic visit and speculum examination. The advent of relatively inexpensive, rapid and accurate HPV DNA testing makes it possible to return screening results from self-sampling very soon after specimen collection, minimizing loss to follow-up. Partial HPV typing provides important risk stratification useful for triage of HPV-positive women. A second “triage” test is often useful to guide management. In lower-resource settings, visual inspection with acetic acid (VIA) is still proposed but it is inaccurate and poorly reproducible, misclassifying the risk stratification gained by primary HPV testing. A deep-learning based approach to recognizing cervical precancer, adaptable to a smartphone camera, is being validated to improve VIA performance. The advent and approval of thermal ablation permits quick, affordable and safe, immediate treatment at the triage clinic of the majority of HPV-positive, triage-positive women.

**Conclusions:**

Overall, only a small percentage of women in cervical screening programs need to attend the hospital clinic for a surgical procedure, particularly when screening is targeted to the optimal age range for detection of precancer rather than older ages with decreased visual screening performance and higher risks of hard-to-treat outcomes including invasive cancer.

## Introduction

Cervical cancer remains a leading female malignancy. Highly efficacious vaccines against human papillomavirus (HPV) and accurate screening methods have been developed; however, in practice, global preventive efforts have lagged behind scientific advances. The COVID-19 (coronavirus disease 2019) pandemic will unavoidably decrease the resources that health systems can devote to cervical cancer prevention. The pandemic, if it persists, threatens to increase morbidity and mortality from many diseases including cervical cancer, due to disruption of clinical and preventive services, and because of delay in implementation of new health initiatives [1].

It is important not to abandon the recent WHO (World Health Organization) call for elimination of cervical cancer. At the same time, given the potential threat of SARS-CoV-2 (severe acute respiratory syndrome coronavirus 2) spread, and limited preventive resources that are now even more strained, those of us devoted to cervical cancer prevention must adapt. The goal of cervical screening is to identify the few women with cervical precancer that can be treated to prevent cancer, while minimizing harm to the great majority. Even an important malignancy like cervical cancer is uncommon, and invasive disease typically takes decades to develop. Less than 5% of women in most populations are at risk of cervical cancer in their lifetime; at a single point in time, only 1% or less of women have precancer.

In this commentary, we propose a cervical screening research and implementation strategy designed to take into account the typically slow natural history of cervical cancer and the severe but hopefully temporary impact of COVID-19. In three companion manuscripts [2–4], we highlight the practical validation of some critical components of the strategy, drawing on recent project results in Asia and Africa.

## Possible cervical screening approach in the COVID-19 era

The main phases of a cervical screening program are 1) primary screening of women in the general population, 2) triage testing of the small minority of women that screen positive to determine need for treatment, and 3) treatment of triage-positive women thought to be at highest risk of precancer or even cancer. In each phase, attention must now be paid to safety in relation to SARS-CoV-2 transmission.

### Primary screening

The new imperatives of the COVID-19 pandemic support self-sampled HPV testing as the primary cervical screening method. HPV DNA testing is the most sensitive cervical screening test, which thereby confers the greatest and longest reassurance when the test is negative [5]. A negative HPV DNA test provides sufficient reassurance that it is worth questioning whether women whose last test was HPV-negative need to return during this phase of the pandemic or are might consider waiting for a better benefit-to-risk timing.

Self-sampling to obtain the specimen for HPV testing yields reassurance equivalent to clinician sampling, and has proven to be quite practical and teachable by simple graphics or animated video presentation, as demonstrated by the accompanying manuscript from Nigeria [4]. A sealed, clean self-sampler could be delivered to women and gathered in a COVID-safe approach.

The advent of relatively inexpensive, rapid and accurate HPV DNA testing, as validated in an accompanying article from the prominent research team in Shenzhen, China [2], makes it possible to return screening results from self-sampling very soon after specimen collection, minimizing loss to follow-up. As shown by the international collaborative effort from Inner Mongolia [3], the combination of self-sampling and rapid HPV testing even permits high-volume, same-day health fair approaches. Such social crowding is now contraindicated but mass screening approaches might be possible again, and important in reaching the huge numbers of unscreened women, once the COVID-19 pandemic is past.

### Triage

HPV testing is sensitive but not specific because most infections will not cause precancer; fewer still will lead to cancer. The risk is dependent on HPV type. Typing of individual HPV types can be incorporated with minimal additional cost into self-sampled HPV testing, providing important risk stratification useful for triage of HPV-positive women. The types of HPV defined as carcinogens vary quite substantially in risk. HPV16 is uniquely carcinogenic. HPV18 and HPV45 are important causes of cervical cancer but tend for reasons that are still not completely understood to cause relatively few diagnosed cases of precancer. The types of HPV related to HPV16, namely, HPV31, HPV33, HPV35, HPV52, and HPV58 are conceptually worth distinguishing from the lower risk, minimally carcinogenic types (HPV39, HPV51, HPV56, HPV59, and HPV68) [6]. Of note, HPV35 is particularly pernicious for African women [7]. Typing would permit screening program planners to concentrate on treatment of the highest risk women, and to focus more accurately on avoiding harm to those at lower risk.

Because even the highest-risk HPV types are common in aggregate at screening ages in high-prevalence settings (> 10% overall), a second “triage” test is useful to guide management. In high-resource settings, reflex cervical cytology or the novel dual-stain immunocytochemical test [8] are currently preferred. In lower-resource settings, visual inspection with acetic acid (VIA) is still proposed but it is inaccurate and poorly reproducible, misclassifying the risk stratification gained by primary HPV testing.

**Fig. 1:**
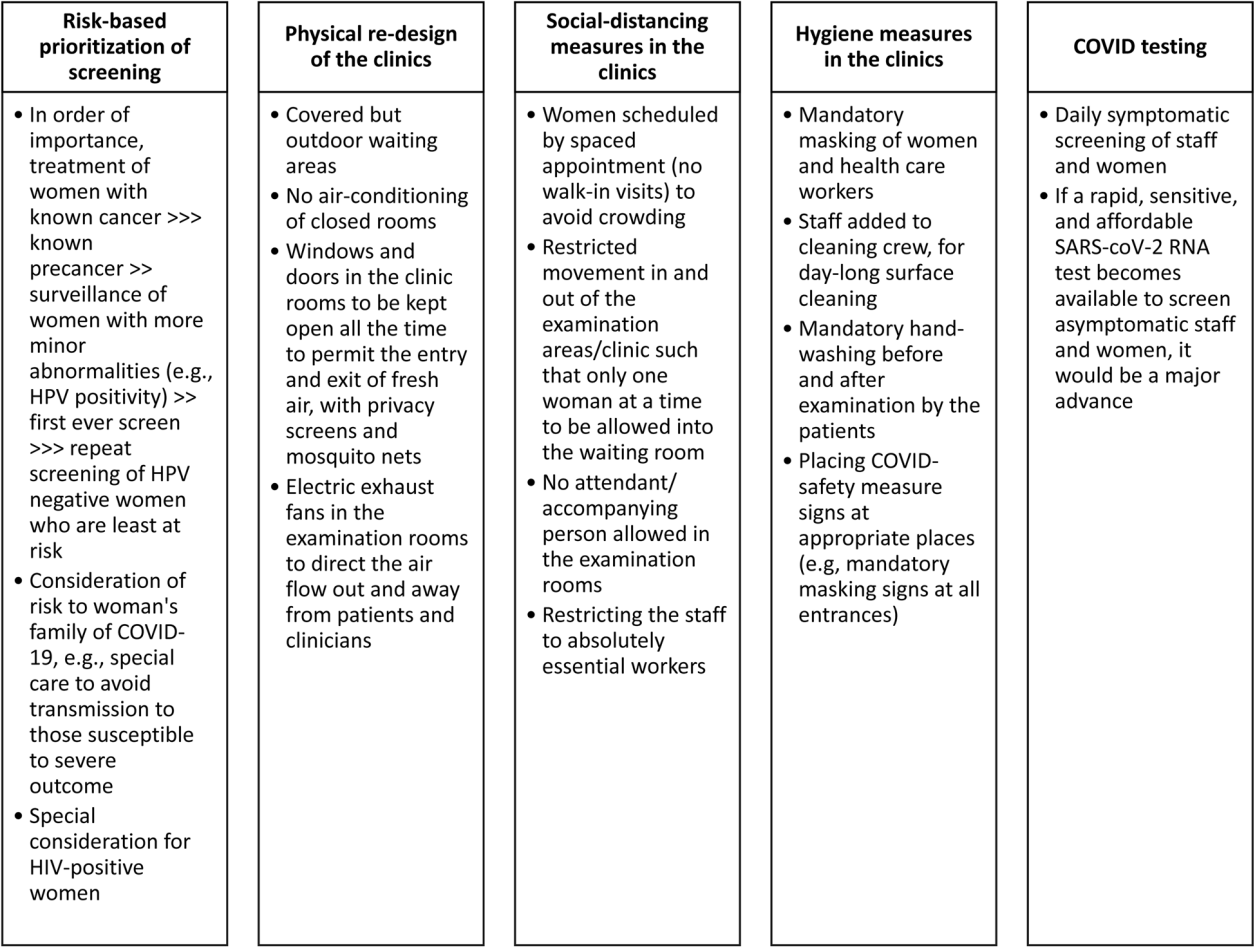
Redesigning out-patient triage/treatment clinic for cervical screening in the COVID-19 era: Example of Ile-Ife, Nigeria

Automated visual evaluation (AVE) is under development as a triage diagnostic technology [9]. This is a deep-learning based approach to recognizing cervical precancer, adaptable to a smartphone camera. It still requires a speculum examination for picture taking by a health worker but, in combination with HPV typing, could provide excellent risk stratification as to which women have precancer and need treatment. Validation and efficacy studies of AVE with and without prior HPV typing are underway. Somewhat earlier in development, DNA methylation assays that reveal the shift of productive HPV infections to precancer might permit molecular triage of the self-sample, identifying only the highest risk women for presentation to a clinic [10]. Triage clinics can be situated away from hospitals or other settings caring for coronavirus-infected patients; this could plausibly decrease risk of viral spread and increase attendance. Moreover, environmental engineering of the clinics to promote pass-through airflow, social distancing, and protective masks, can be introduced into clinical management of screen-positive women. Added costs to promote safety are worth considering at the current time, although all recognize that the expense is not supportable in low-resource regions indefinitely. As an illustration, in the sidebar, safety steps planned in the next phase of the Nigerian project are outlined (Fig. 1). The practicality of this effort however will depend not only on cost, but also on the perception of safety by the end-users. Long-term sustainability will depend on not needing such measures indefinitely, as the pandemic is brought under control.

### Treatment

**Fig. 2:**
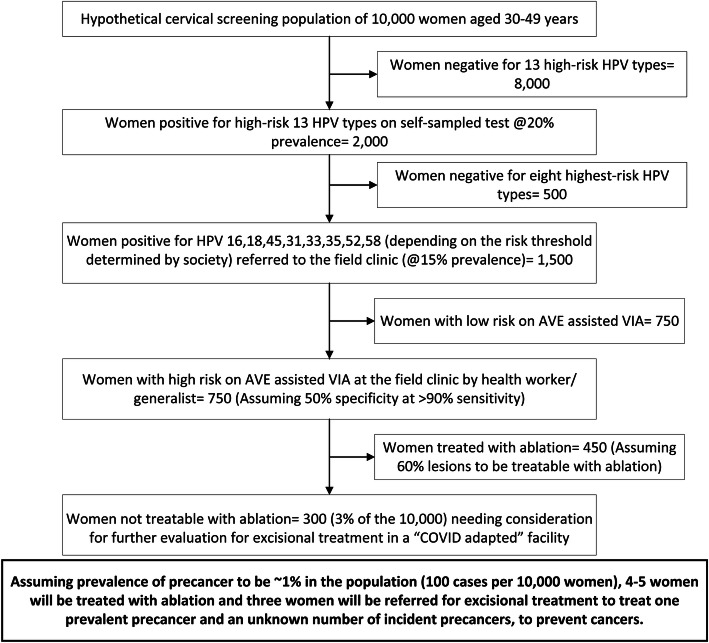
Effect of HPV based screening followed by triage to reduce referral in a hypothetical population

The advent and approval of thermal ablation permits quick, affordable and safe, immediate treatment at the triage clinic of the majority of HPV-positive, triage-positive women. As a part of AVE, deep-learning algorithms are in development that will assist health workers in the judgment of whether a lesion can be ablated or whether referral for excision is required. For those women needing an excisional large-loop excision of the transformation zone (LLETZ) procedure, mobile battery-operated LLETZ units and innovative electrode designs are now available to permit safer local performance, and possible distancing of those treatments from hospitals devoted to COVID-19 care. Overall, only a small percentage of women in cervical screening programs need to attend the hospital clinic for a surgical procedure (Fig. 2), particularly when screening is targeted to the optimal age range for detection of precancer rather than older ages with decreased visual screening performance and higher risks of hard-to-treat outcomes including invasive cancer.

It is imperative to keep in mind that it is the treatment of screen-detected precancer and not the screening of precancer that prevents the development of cancer. In this context, AVE and other screening methods must be backed up by a tracking system to minimize loss to follow up from screening to triage to treatment, particularly in low-resource settings without any existing electronic medical records systems. The establishment of a routine screening program, with repeated surveillance for HPV negative and low-risk HPV positive women, is yet another challenge in low-resource settings that is likely to persist even past the COVID pandemic. A risk threshold for treatment based on a combination of HPV types, AVE score, and other triage tests needs to be chosen very carefully in such settings given the risk of exposure to COVID, availability of resources, and the reality of once or twice in a lifetime access to screening for many women relying on the public sector.

## Conclusions

The COVID-19 pandemic motivates the transition away from reliance on speculum examinations for screening. It might logically be the right time to move away from such methods including the venerable Pap test, in favor of reliable and accurate HPV-based technologies based on self-sampling. Affordability remains, however, a real and important constraint. The affordability of screening is made more challenging by new safety considerations that limit the social crowding implicit in high-throughput lower-cost approaches. The need for such measures will ease as the pandemic eventually ends, but the current emergency mandates that we push past the inertia of current practices. Primary screening is “elective”, in that the great majority of women are not at immediate risk. In restarting existing screening efforts, it makes sense to prioritize women already known to have precancer in need of treatment, paying intermediate attention to those with lesser abnormalities, and to consider very cautiously for now the net benefit of routine screening for low-risk women previously screened HPV-negative.

## Data Availability

Not applicable.
